# Improvement of uridine production of *Bacillus subtilis* by atmospheric and room temperature plasma mutagenesis and high-throughput screening

**DOI:** 10.1371/journal.pone.0176545

**Published:** 2017-05-04

**Authors:** Xiaoguang Fan, Heyun Wu, Guoliang Li, Hui Yuan, Hongchao Zhang, Yanjun Li, Xixian Xie, Ning Chen

**Affiliations:** 1 National and Local United Engineering Lab of Metabolic Control Fermentation Technology, Tianjin University of Science and Technology, Tianjin, P. R. China; 2 Key Laboratory of Microbial Engineering of China Light Industry, Tianjin University of Science and Technology, Tianjin, China; 3 College of Biotechnology, Tianjin University of Science and Technology, Tianjin, P. R. China; National Renewable Energy Laboratory, UNITED STATES

## Abstract

In the present study, a novel breeding strategy of atmospheric and room temperature plasma (ARTP) mutagenesis was used to improve the uridine production of engineered *Bacillus subtilis* TD12np. A high-throughput screening method was established using both resistant plates and 96-well microplates to select the ideal mutants with diverse phenotypes. Mutant F126 accumulated 5.7 and 30.3 g/L uridine after 30 h in shake-flask and 48 h in fed-batch fermentation, respectively, which represented a 4.4- and 8.7-fold increase over the parent strain. Sequence analysis of the pyrimidine nucleotide biosynthetic operon in the representative mutants showed that proline 1016 and glutamate 949 in the large subunit of *B*. *subtilis* carbamoyl phosphate synthetase were of importance for the allosteric regulation caused by uridine 5′-monophosphate. The proposed mutation method with efficient high-throughput screening assay was proved to be an appropriate strategy to obtain uridine-overproducing strain.

## Introduction

Uridine and its nucleotide derivatives play an important role in the biochemical and physiological processes including the synthesis of DNA, RNA, membrane constituents and glycosylation [1]. Furthermore, uridine has been increasingly used as precursors for antivirus and antitumor drugs in pharmaceutical industry [2, 3]. Chemical synthesis is the commercial method for uridine production by the condensation reaction of uracil and D-ribose, however, the expensive raw materials increase the production cost. Other methods such as enzymatic decomposition of ribonucleic acid and salvage synthesis from uracil or orotic acid are also used for uridine production [4]. However, the complicated process such as nucleoside separation and enzyme production limits their industrial-scale application.

*Bacillus subtilis* is able to synthesize uridine 5′-monophosphate (UMP) through *de novo* pyrimidine biosynthesis. UMP can be further converted to uridine by dephosphorylation using nucleotide phosphoesterase. However, the intracellular synthesis of uridine is strictly controlled at a transcriptional level. It has been confirmed that 10 genes encoding all enzymes involved in the *de novo* synthesis of UMP lie in a signal coordinately related *pyr* operon [5]. The expression of the *pyr* operon is down regulated by the PyrR regulatory protein which is encoded by the first gene of the *pyr* operon [6, 7]. The combination of PyrR to the binding loop of *pyr* mRNA can be activated by sufficient amount of UMP [8, 9]. In addition, the first enzyme in the pyrimidine biosynthetic pathway, carbamoyl phosphate synthetase (CPSase), which catalyzes the synthesis of carbamoyl phosphate from bicarbonate, ammonia, and two molecules of ATP, is the rate-limiting enzyme and subject to feedback inhibition by UMP [10, 11]. Therefore, relieving the feedback regulation caused by UMP would be significant for improving the uridine production of *B*. *subtilis*.

In a previous study, we cooperated with Tianjin University to modify some key genes and operons related to the pyrimidine nucleotide biosynthesis in *B*. *subtilis* 168 [12]. The resultant strain *B*. *subtilis* TD 12np (*trpC2*, *ΔaraR*::*Para-neo*^*R*^, *Δcdd*, *Δhom*, *ΔpyrR*, *ΔnupC-pdp*) accumulated 1.2 g/L uridine in shake-flask fermentations. Although the *pyrR* gene that responsible for the transcription regulation of the *pyr* operon was deleted to reduce the feedback repression, the negative cooperative effect of UMP on CPSase activity was still unsolved due to the lack of information on the regulatory site of *B*. *subtilis* CPSase. It has been reported that in *Escherichia coli*, the inhibitor UMP binds in the carboxy-terminal domain of the large subunit of CPSase, a domain of ~15 kDa [13, 14]. Some amino acid residues (serine 948, threonine 977, lysine 992) located in this domain are essential for allosteric regulation [15–17]. However, the UMP binding sites of *B*. *subtilis* CPSase are still unclear.

Traditional strategies to enhance the resistance and production of wild-type strains includes adaptive laboratory evolution and random mutagenesis by nitrosoguanidine and ultraviolet treatment [18, 19]. The challenge is to develop efficient screening methods to acquire mutants with desired phenotypes. Although combining cytidine deaminase (CDA) and indophenol method have been performed in 96-well microplate for the high-throughput screening of cytidine producing strains, the two-step reactions and purified CDA required in the process are expensive and time-consuming [20]. Recently, a novel breeding strategy of atmospheric and room temperature plasma (ARTP) mutagenesis has been successfully applied to the microbial production of lactic acid, arachidonic acid and ε-poly-L-lysine [21–23]. So in the present study, novel ARTP irradiation-induced mutation was carried out on *B*. *subtilis* TD 12np to improve the production of uridine. A simple screening procedure was established using both resistant plates and 96-well microplates to select the ideal mutants with diverse phenotypes. The representative mutants were examined by shake-flask and fed-batch fermentations. Sequence analysis of the pyrimidine nucleotide biosynthetic operon in different mutants was performed to detect the potential regulatory sites of CPSase in *B*. *subtilis*.

## Material and methods

### Parent strain

The parent strain *B*. *subtilis* TD 12np (*trpC2*, *ΔaraR*::*Para-neo*^*R*^, *Δcdd*, *Δhom*, *ΔpyrR*, *ΔnupC-pdp*) was previously constructed in Tianjin university [12]. This strain was derived from *B*. *subtilis* 168 by modification of a series of genes involved in pyrimidine nucleoside biosynthesis.

### Medium

Optimized medium was used for strain screening and uridine fermentation. Resistant plate medium contained 10 g/L glucose, 3 g/L (NH_4_)_2_SO_4_, l g/L K_2_HPO_4_, 3 g/L casamino acids, 0.1 g/L MgSO_4_·7H_2_O, 2 mg/L ZnSO_4_, 2 mg/L MnSO_4_, 0.1 mg/L thiamine, 0.1 mg/L biotin, 0.1 mg/L threonine, 0.1 mg/L methionine, 0.1 mg/L isoleucine, 0.1 mg/L tryptophan and appropriate concentrations of UMP analogues (2-thiouracil, 6-azauracil, and 5-fluorouracil). Microplate medium contained 100 g/L glucose, 5 g/L yeast extract, 10 g/L tryptone, 15 g/L NaNO_3,_ 1 g/L KH_2_PO_4_, 5.2 g/L K_2_HPO_4_, 1 g/L MgSO_4_·7H_2_O and 1 g/L sodium citrate. The component of seed medium was the same as microplate medium except for the glucose concentration (40 g/L). Fermentation medium contained 40 g/L glucose, 5 g/L yeast extract, 5 g/L (NH_4_)_2_SO_4_, 20 ml/L corn steep liquor, 1 g/L KH_2_PO_4_, 5.2 g/L K_2_HPO_4_, 5 g/L MgSO_4_·7H_2_O, 10 g/L sodium citrate, 20 g/L glutamate, 20 g/L area, 1 g/L CaCl_2_, 0.02 g/L ZnSO_4_·7H_2_O and 0.02 g/L MnSO_4_·H_2_O. The pH of all medium was adjusted to 7.0 with 5 M NaOH before sterilization. Glucose was sterilized separately at 115°C for 15 min.

### Process of ARTP mutagenesis

ARTP mutation breeding system [24] was purchased from Si Qing Yuan Biotechnology Co. Ltd, Beijing, China. In each ARTP mutagenesis, 1 mL cells suspension of parent strain was inoculated in 30 mL LB medium and cultured for 6 h. The active cells were harvested, washed twice with sterile saline and diluted to 10^7^–10^8^ CFU/mL. Then 10 μL fresh prepared cells suspension were poured into a sterilized stainless steel plate (1 cm diameter) and exposed to the plasma jet. The operating parameters were as follows: pure helium was used as the plasma working gas; radio frequency power input was 100 W; distance between the plasma torch nozzle exit and the sample plate was 2 mm; temperature of the plasma jet was below 30°C; Treatment time of ARTP was set as 25 s. After each treatment, the sample plates were transferred into the centrifuge tubes containing 1 mL sterile saline to wash the cells off. The suspension was collected for screening steps.

### High-throughput screening of target mutants

After ARTP mutagenesis, 100 μL cells suspension was spread on a resistant plate containing 200 mg/L 2-thiouracil and cultivated at 36°C for 24 h. A library of mutants with rapid growth was constructed, and the mutants were transferred to a 96-well microplate with 400 μL LB culture medium each well. The microplates were cultivated at 36°C, 200 rpm for 12 h. 40 μL of the culture in each well was then inoculated into the corresponding well of another 96-well microplate with 360 μL microplate medium each well and cultivated at 36°C, 200 rpm for 30 h. Uridine concentrations in each culture were measured at 270 nm using microplate reader (Tecan Austria GmbH, Austria) after centrifugation and dilution. The uridine content (g/L) was evaluated according to the following equation (S1 Fig).

$$\mathrm{Uridine}\;\mathrm{content}=\left(OD_{270}–0.01415\right)/0.01786\times 0.1$$ (1)

**Fig 1:**
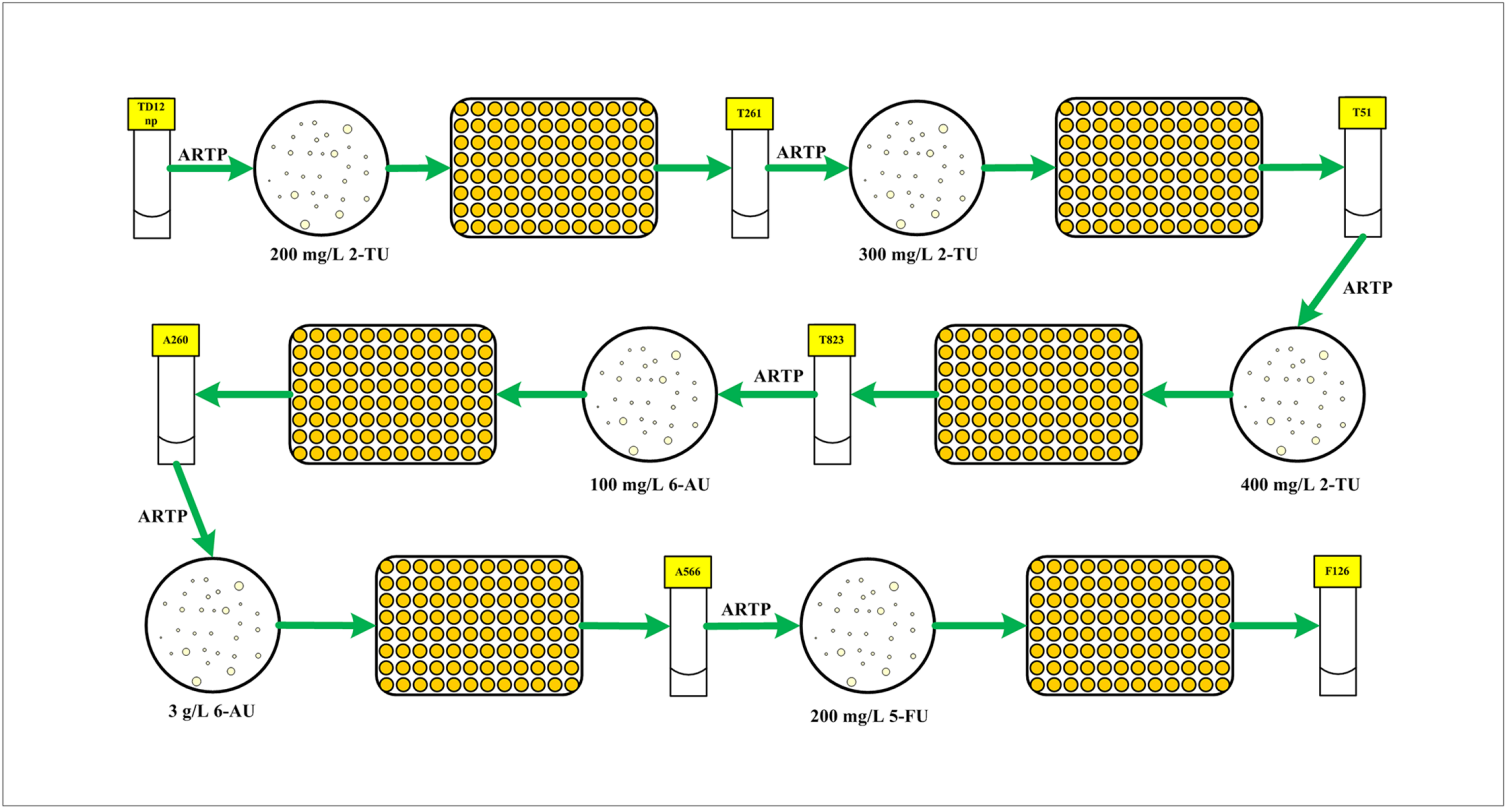
Procedure of high-throughput screening of *B*. *subtilis* mutants generated by ARTP. 2-TU represents 2-thiouracil; 6-AU represents 6-azauracil; 5-FU represents 5-fluorouracil.

The mutant with the highest uridine content was selected and used for the next round of ARTP mutagenesis. Three successive rounds of ARTP mutagenesis were performed under different concentrations of 2-thiouracil (200, 300 and 400 mg/L) by the same method. After that, 6-azauracil and 5-fluorouracil were also used as the UMP analogues for tolerant strain screening. The final concentrations of 6-azauracil and 5-fluorouracil in the resistant plate were raised to 3 g/L and 200 mg/L after two and one rounds of ARTP mutagenesis, respectively. The detailed procedures of rounds with different UMP analogues are illustrated in Fig 1.

### Cultivation of target mutants in shake-flask and 5-L fermenter

To test the fermentation performance and genetic stability, 2 mL cells suspension of the target mutants was transferred into a 500 mL shake-flask containing 30 mL microplate medium and incubated at 36°C, 200 rpm on a rotating shaker for 30 h cultivation. The pH was kept to 7.0 by addition of 4 M ammonium hydroxide using a micro-injector according to the color change of the indicator of phenol red in the culture. Genetic stability of the target mutants was also examined by a series of sub-cultures in the shake-flask for the uridine detection.

To evaluate the potential advantage of the target mutants, fed-batch fermentation was performed in a 5-L fermenter (Baoxing, Shanghai, China) containing 2.7 L fermentation medium. 300 mL seed culture from shake-flask cultivation was transferred into the fermenter and the reaction condition was set as follows: The pH was automatically controlled at 7.0 by addition of 5 M NaOH solution and sterilized soybean meal hydrolysate. Dissolved oxygen was maintained at ~30% of air saturation by variation of the stirrer speed and the aeration rate. The temperature was kept constantly at 36°C. When the glucose in fermentation medium was consumed, a mixed solution containing 800 g/L glucose and 60 g/L yeast extract was added automatically at an appropriate rate to maintain the residual glucose at ~5 g/L.

### Analytical methods

Cell growth was monitored by measuring the absorbance at 600 nm (OD_600_). OD_600_ was converted to dry cell weight by a calibration curve. Glucose concentration was measured by an SBA-40E biological sensor (Shandong Academy of Sciences, Shandong, China). The uridine concentration in shake-flask and 5-L fermenter was quantified by isocratic HPLC (Thermo Scientific UltiMate 3000) using a Gemini C18 column (Phenomenex, USA). Acetonitrile/water mixture (2:98 v/v) was used as the mobile phase at a flow rate of 1 mL/min. The wavelength of UV detector and temperature of column oven were 270 nm and 30°C, respectively.

## Results and discussion

### Design of mutagenesis and high throughput screening

The parent strain *B*. *subtilis* TD 12np (*trpC2*, *ΔaraR*::*Para-neo*^*R*^, *Δcdd*, *Δhom*, *ΔpyrR*, *ΔnupC-pdp*) was mutated by a novel mutation method of ARTP. For generating an appropriate mutant library, the lethality rate was detected under different treatment times (10, 30, 45, 60 and 75 s) of plasma jet and evaluated based on the following equation: $\mathrm{Lethality}\;\mathrm{rate}\;(\%)=\frac{U\text{-}T}{U}\times 100\%$ (U is the total count of control colonies grown on the LB solid medium and T is the total count of survival colonies grown on the LB solid medium). When the cells suspension was treated with ARTP for 10, 30, and 45 s, the lethality rates increased to 60.7%, 92.7% and 99.5% respectively (S2 Fig). When the treatment time was more than 60 s, no cells survived. According to the literature, higher positive mutation rates could be realized when the lethality rate was around 80% [21, 25]. Therefore, a treatment time of 25 s was used for mutagenesis.

**Fig 2:**
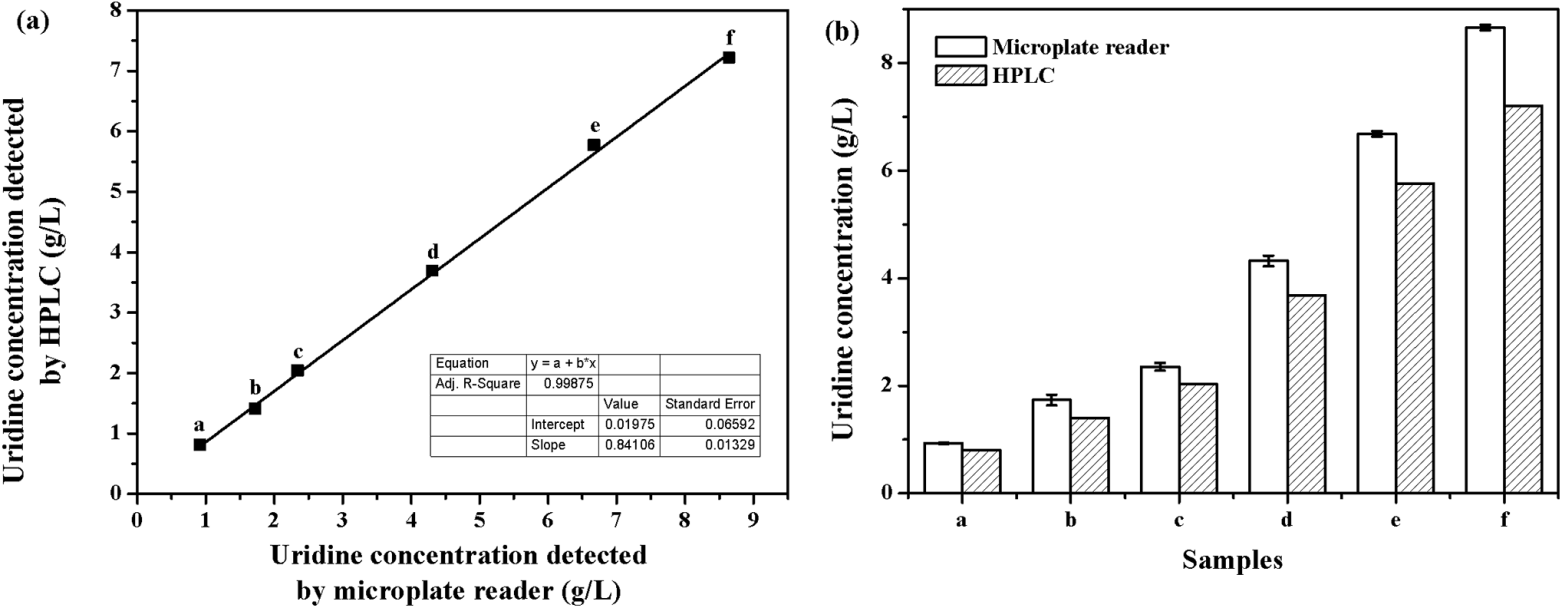
Comparison of uridine concentration detected by microplate reader and HPLC. (a) Correlation coefficient of the two methods; (b) Measured values of uridine concentration in microplate. Each plot represents the average of three samples.

Selection for antimetabolite resistance has proven to be successful in development of nucleoside fermentations. Cytidine-producing mutants derived from a *B*. *subtilis* cytidine-deaminase-deficient strain could resist 500 mg/L 5-fluorocytidine and accumulate 10.4 g/L cytidine [26]. Further mutants with resistance to 3-deazauracil increased cytidine titer to 14.2 g/L [27]. Although positive mutants can be preliminary selected by toxic nucleoside analogues, the production performance of mutants still need to be tested by HPLC after shake-flask fermentation. That means the numbers of mutants that can be analyzed at once are limited. Therefore, development of rapid and accurate screening methods is necessary for understanding the behavior of mutants [28]. Considering the main product of *B*. *subtilis* TD 12np was uridine, and the *pdp* gene that related to the formation of uracil and D-ribose-1-phosphate has been deleted, the uridine concentration of fermentation broth in the 96-well microplate was directly detected by microplate reader at a wavelength of 270 nm (OD_270_ nm). To reduce the effect of abnormal growth on uridine titer, the mutants grown in the resistant plate were firstly transferred into 96-well microplate with LB medium for cultivation, and then accurately inoculated into another 96-well microplate with optimized microplate medium for uridine fermentation. As shown in Fig 2, the values of uridine concentration detected by microplate reader and HPLC fit well and the correlation coefficient reached to 0.9987. The slightly higher value obtained by microplate reader was mainly due to a small amount of cytidine and uracil formed during the fermentation.

### Selection of enhanced uridine-producing mutants

**Fig 3:**
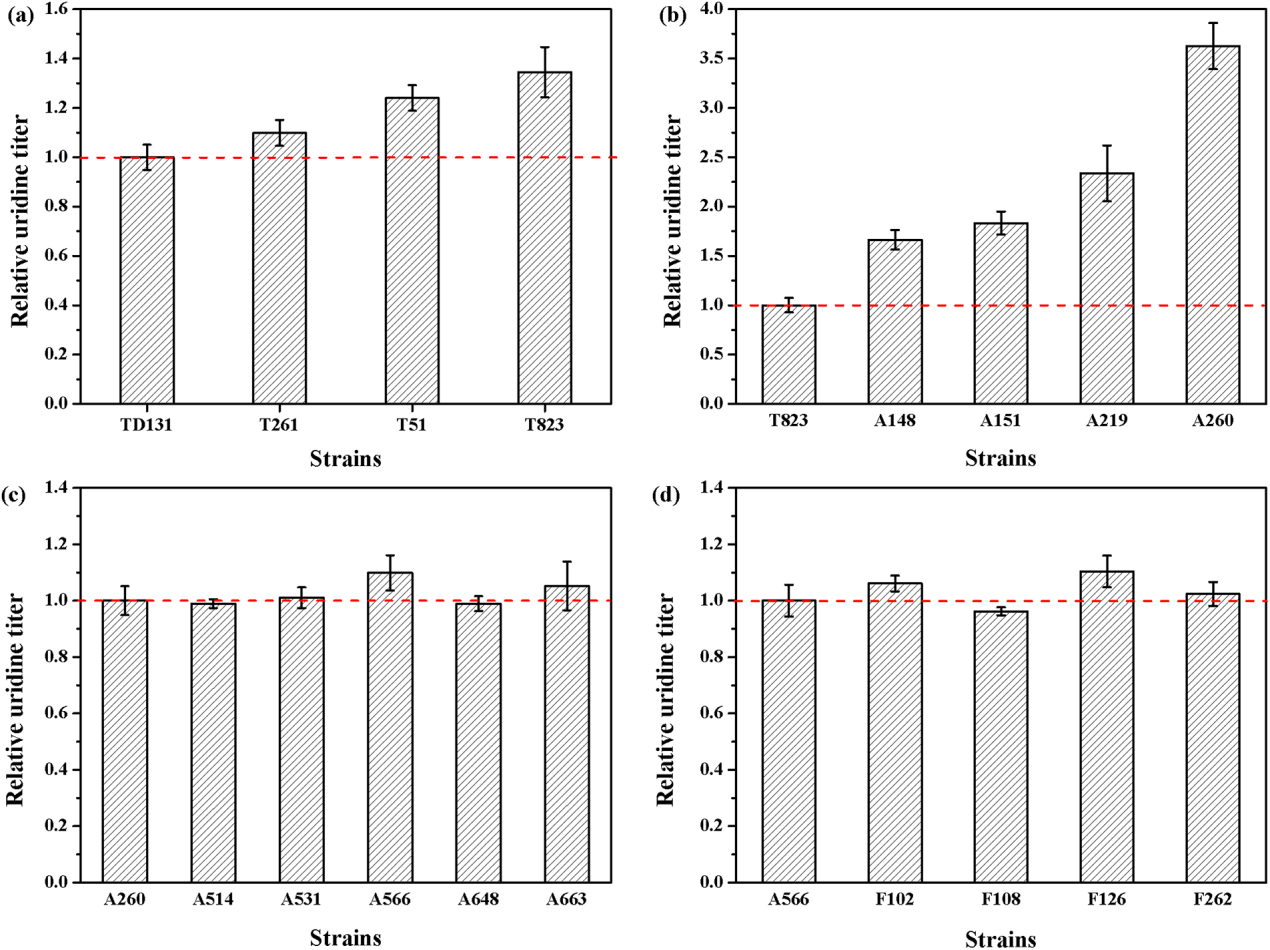
Relative uridine titer of representative mutants after different rounds of ARTP mutagenesis. (a) Mutants isolated from 200 mg/L, 300 mg/L and 400 mg/L 2-thiouracil-resistance plate; (b) Mutants isolated from 100 mg/L 6-azauracil-resistance plate; (c) Mutants isolated from 3 g/L 6-azauracil-resistance plate; (d) Mutants isolated from 200 mg/L 5-fluorouracil-resistance plate.

In *B*. *subtilis*, *pyrR* gene encodes regulatory protein PyrR which regulates transcriptional attenuation of *pyr* operon by binding in a uridine nucleotide-dependent manner to specific sites on *pyr* mRNA [29, 30]. The deletion of *pyrR* gene in the parent strain TD 12np resulted in enhanced expression of all six UMP biosynthetic enzymes. However, the activity of CPSase, the first enzyme of the pyrimidine biosynthetic pathway still suffered from feedback inhibition caused by UMP. As shown in Table 1, the growth of parent strain TD 12np was strongly inhibited by uridine analogues at a very low concentration. To release the feedback inhibition and enhance the uridine yield, different concentrations of 2-thiouracil (from 200 to 400 mg/L, see Fig 1) were firstly used for resistance screening after three rounds of ARTP mutation. Mutant T261, T51, T823 with increasing 2-thiouracil tolerance and uridine production was selected in each round by microplate reader (S3 Fig). Unfortunately, none of the mutants showed a 30% enhancement in uridine titer compared with the parent strain TD 12np (Fig 3A). It suggested that feedback inhibition has not been completely relieved using 2-thiouracil as the UMP analogue.

**Table 1:** Effect of UMP analogues on the growth of parent and mutant strains.

UMP analogues	Minimum lethal concentration, MIC_100_^a^ (mg/L)							
	TD12np	T823	A219	A260	A566	F126	TD 12np-*pyrAB* ^P1016L^	TD 12np-*pyrAB* ^E949*^
**2-thiouracil**	150	400	3000	3500	4000	4000	2300	2600
**6-azauracil**	100	150	3500	3000	4500	4500	2700	2500
**5-fluorouracil**	0.08	1	100	160	200	2500	60	80

^a^MIC_100_ represents concentration of UMP analogues that yields 100% inhibition.

Other UMP analogues, such as 6-azauracil (from 100 mg/L to 3 g/L) and 5-fluorouracil (200 mg/L), were chosen as selective markers for the following resistance screening procedure (S4 and S5 Figs). Some positive mutations which appeared in the resistant medium containing 100 mg/L 6-azauracil showed remarkable increase in uridine production. The uridine titer of mutant A219 and A260 was 2.3- and 3.6-fold higher than the initial strain T823 (Fig 3B). When increasing 6-azauracil concentration up to 3 g/L, only 10% improved uridine titer was achieved in the mutant A566 compared to the initial strain A260 (Fig 3C). When 200 mg/L 5-fluorouracil was adopted, the improvement of uridine titer of mutant F126 was still less than 10% (Fig 3D). These results indicated that feedback inhibition has been successfully relieved during the previous screening with 100 mg/L 6-azauracil.

### Characterization of the representative mutants

After screening the mutant library, representative mutants were selected for characteristics. As shown in Table 2, mutant A219, A260, A566 and F126 accumulated 3.1, 4.7, 5.2, 5.7 g/L uridine respectively, which was 2.3-, 3.6-, 3.9- and 4.4-fold higher than the parent strain TD 12np. Meanwhile, all the mutants showed similar final biomass but better tolerance to the UMP analogues. The minimum growth inhibitory concentration mutant by 2-thiouracil, 6-azauracil and 5-fluorouracil towards mutant F126 was increased to 4, 4.5 and 2.5 g/L, respectively (Table 1). However, it is interesting to note that the improvement of uridine production was only closely related with the resistance to 6-azauracil, suggesting that the enhanced tolerance to high concentration of 2-thiouracil and 5-fluorouracil was probably caused by defection in the uptake of toxic analogues rather than the alteration in the activities of UMP biosynthetic enzymes. In addition, all the mutants showed no significant increase in the cytidine and uracil titer (Table 2), indicating that the resistance to UMP analogues had no effect on the activities of cytidine biosynthetic enzymes and the efficient screening assay established in this study was suitable for obtaining uridine-overproducing strain.

**Table 2:** Pyrimidine nucleoside produced by parent and mutant strains in 30 h shake-flask fermentation.

Strain	Selection plate	Biomass (g/L)	Uridine titer (g/L)	Cytidine titer (g/L)	Uracil titer (g/L)
**TD12np**	Wild	7.21±0.71	1.305±0.095	0.243±0.016	0.281±0.026
**T823**	2-TU^R^ (400 mg/L)	6.84±0.37	1.347±0.103	0.242±0.018	0.278±0.021
**A219**	2-TU^R^ (400 mg/L), 6-AU^R^ (100 mg/L)	6.27±0.51	3.051±0.368	0.302±0.055	1.230±0.080
**A260**	2-TU^R^ (400 mg/L), 6-AU^R^ (100 mg/L)	6.04±0.57	4.730±0.305	0.384±0.033	0.876±0.075
**A566**	2-TU^R^ (400 mg/L), 6-AU^R^ (3 g/L)	6.13±0.34	5.150±0.29	0.332±0.043	0.855±0.063
**F126**	2-TU^R^ (400 mg/L), 6-AU^R^ (3 g/L), 5-FU^R^ (200 mg/L)	6.15±0.62	5.734±0.293	0.250±0.030	0.105±0.015

2-TU^R^ represents 2-thiouracil resistance; 6-AU^R^ represents 6-azauracil resistance; 5-FU^R^ represents 5-fluorouracil resistance.

**Fig 4:**
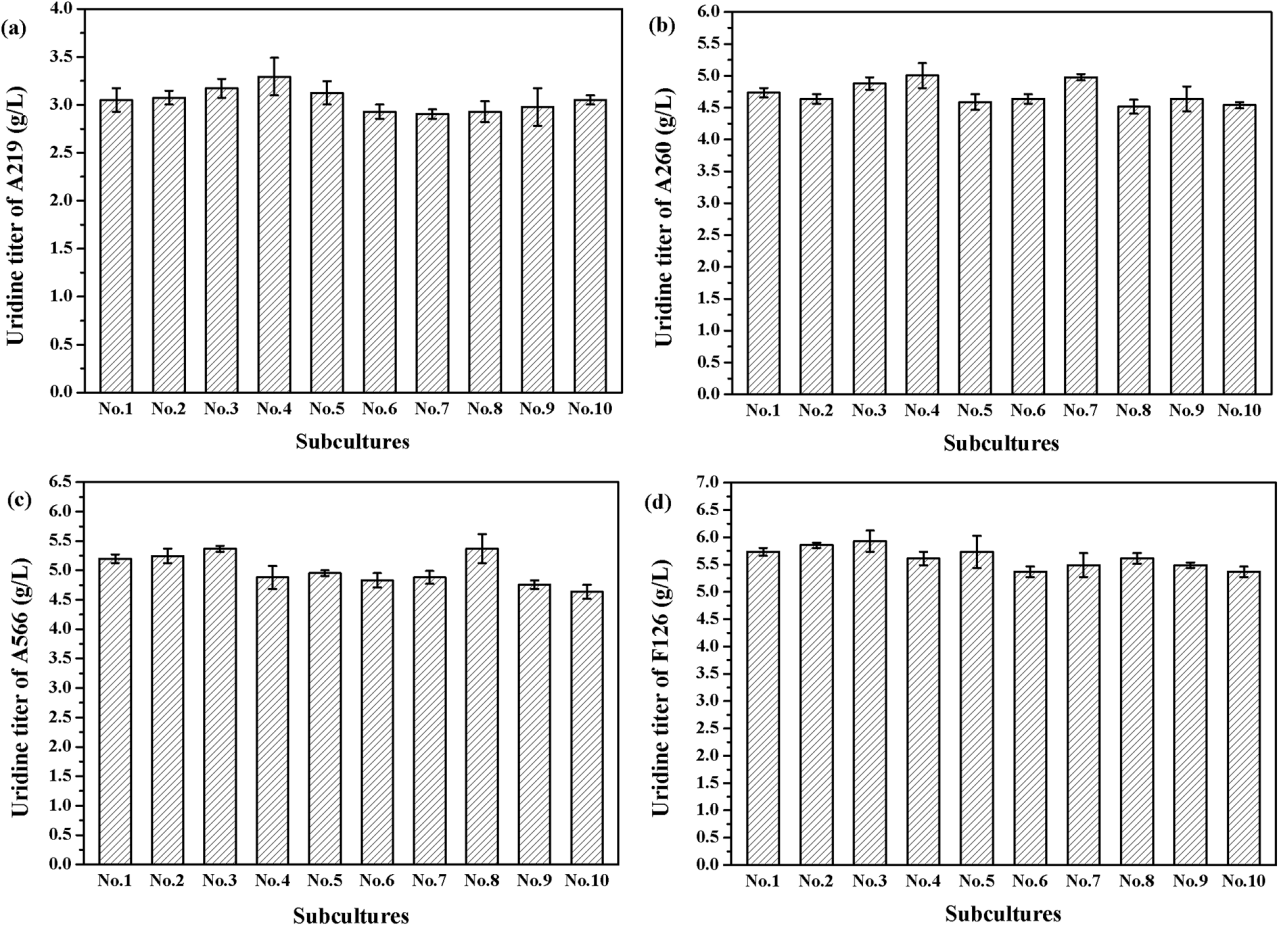
Stability test of mutant strains in ten batches sub-cultures. (a) Mutant A219; (b) Mutant A260; (c) Mutant A566; (d) Mutant F126.

The genetic stability of mutant A219, A260, A566 and F126 in successive sub-cultures was evaluated in 30 h shake-flask fermentation. As shown in Fig 4, the uridine titer of four mutants remained stable even after ten generations, proving that ARTP was effective and genetically stable for improving the diverse traits of bacteria, including the enzyme activities and biochemical productivities [31].

### Performance of the representative mutants in fed-batch fermentation

**Fig 5:**
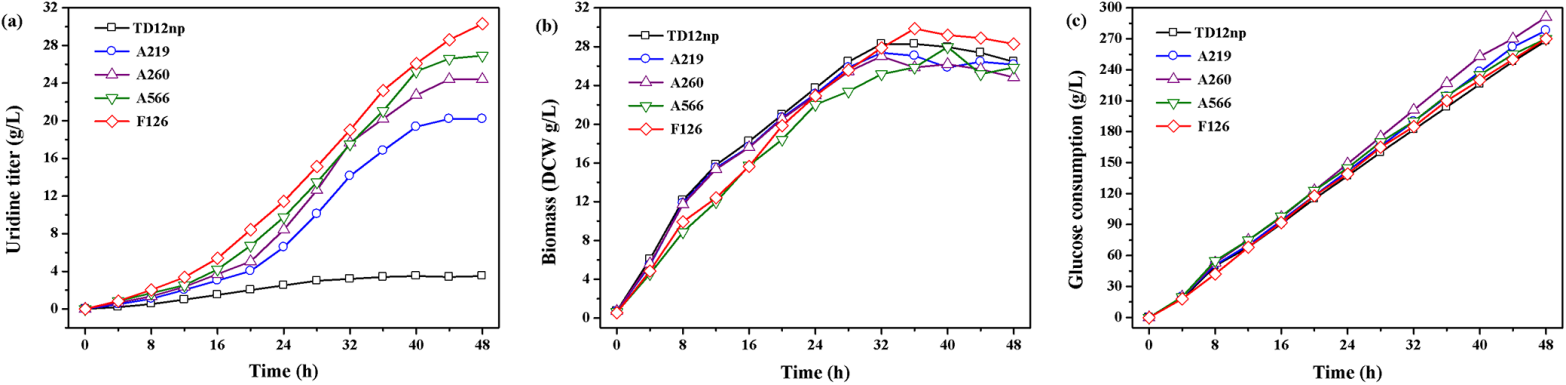
Time course of uridine production by mutant A219, A260, A566, F126 and parent strain TD12np in 48 h fed-batch fermentation. (a) Uridine titer; (b) Biomass; (c) Glucose consumption. Each plot represents the average of three samples.

Fed-batch fermentation has been proven to be an effective strategy for enhancing adenosine production [32]. So it was carried out in this study to further investigate the fermentation characteristics of mutants A219, A260, A566 and F126. As shown in Fig 5, time profiles of mutants and parent strain exhibited the same tendency in terms of cell growth and glucose consumption. Mutant F126 accumulated the highest uridine level of 30.3 g/L in the 48 h fed-batch fermentation, which was 8.7-fold higher than the parent strain TD12np. Meanwhile, the specific uridine production rate was apparently positively correlated with the growth rate under glucose-limited conditions (residue glucose concentration was controlled at about 5 g/L) and was thus coupled to growth. Relative lower uridine production rate was obtained under the conditions of high growth rate (0–20 h), while, uridine accumulation was carried out only at later stage of fed-batch fermentation. It has been demonstrated that *B*. *subtilis* has a high capacity for generating excess NADPH and pentose precursors via the oxidative branch of the HMP pathway at low growth rate compared to other bacteria [33, 34]. Therefore, the significant improvement of uridine titer and yield in mutants (Table 3) was not due to the excess supply of ribose 5-phosphate precursors and cofactors but the increase of metabolic intensity of pyrimidine biosynthesis. In addition, because *B*. *subtilis* devotes a relatively high portion of its energy supply to maintenance metabolism at low growth rate [35], the conversion rates of glucose to uridine of all the mutants were limited.

**Table 3:** Fermentation parameters of parent and mutant strains in 48 h fed-batch fermentation.

Strain	Biomass (g/L)	Glucose consumption (g/L)	Uridine titer (g/L)	Uridine yield (g/g glucose)	Productivity (g/(L× h))
**TD12np**	24.8±2.0	268±35	3.5±0.5	1.3±0.1	0.07±0.01
**A219**	25.8±2.3	278±28	20.2±1.8	7.2±0.4	0.42±0.03
**A260**	28.3±2.4	291±33	24.4±2.2	8.4±0.4	0.50±0.03
**A566**	26.4±2.8	270±26	26.9±3.0	9.9±0.6	0.56±0.04
**F126**	26.1±2.5	270±29	30.3±2.5	11.2±0.8	0.63±0.04

### Sequence analysis of *pyr* operon of the representative mutants

**Fig 6:**
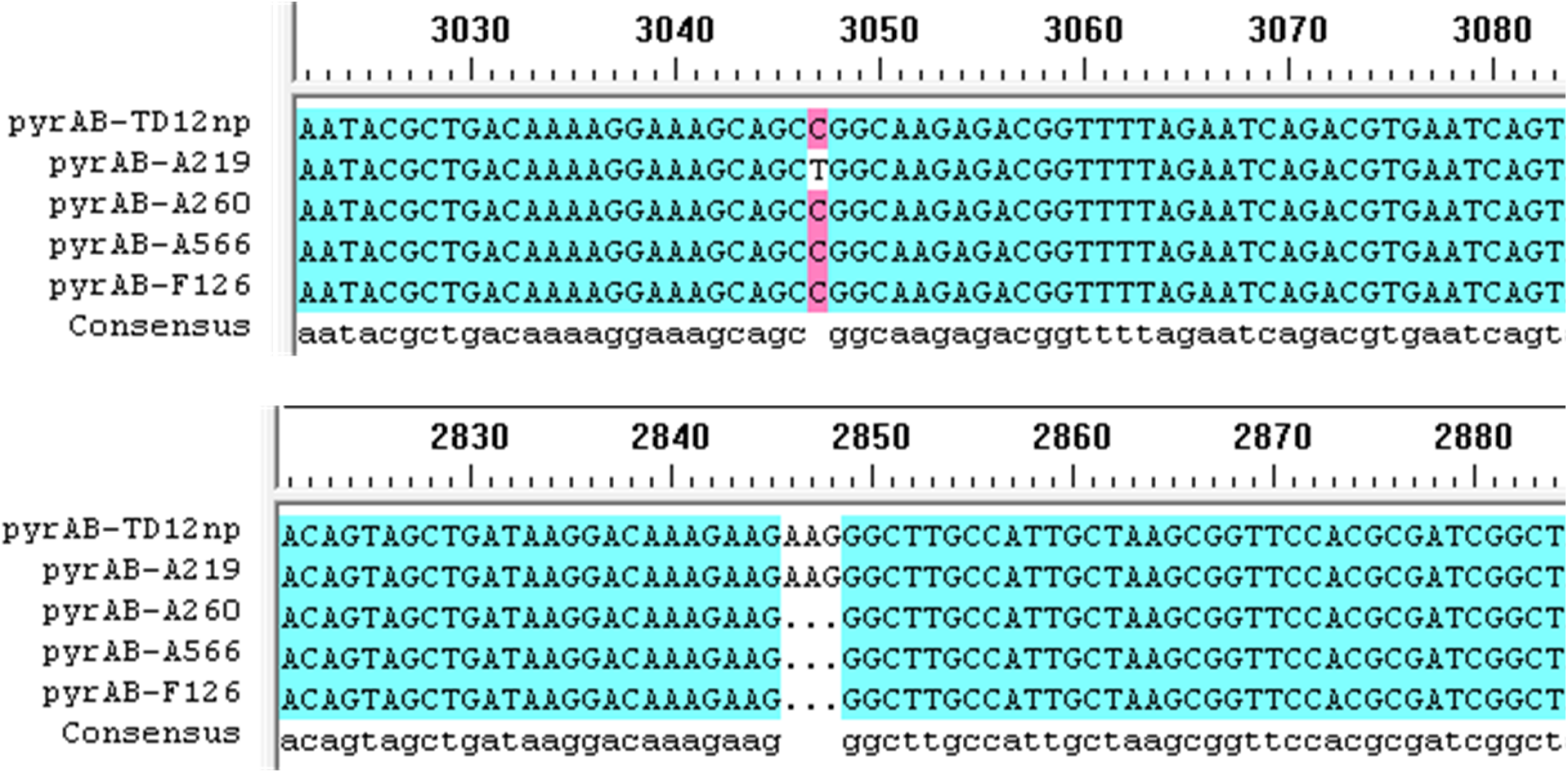
Multiple-sequence alignment of *pyrAB* gene in mutant A219, A260, A566, F126 and parent strain TD12np.

The entire pyrimidine biosynthetic (*pyr*) genes are clustered on a 12.5-kilobase segment of *B*. *subtilis* chromosome [36]. Sequencing results of the *pyr* cluster of the mutants showed that mutations were generated only in the *pyrAB* gene. In *B*. *subtilis*, the pyrimidine repressible CPSase is encoded by two cistrons, *pyrAA* and *pyrAB*, which are homologous to the *E*. *coli carA* and *carB* genes [5]. The smaller subunit encoded by *pryAA* gene is responsible for the hydrolysis of glutamine, whereas the larger subunit encoded by *pryAB* gene harbors the binding sites for substrates and allosteric ligand and catalyzes the formation of carbamoyl phosphate [37]. As shown in Fig 6, the 3047th base of *pyrAB* gene was changed from cytimidine to thymine in mutant A219 and three consecutive bases from position 2846 to 2848 of *pyrAB* gene was missing in mutant A260, A566 and F126. Base pair C3047T resulted in residue replacement (P1016L) and base pair 2846AAG2848 deletion resulted in open reading frame shift (E949*).

The domain from residue 933 to 1073 in the large subunit of *E*. *coli* CPSase is responsible for the binding and allosteric regulation by UMP and inosine 5′-monophosphate (IMP) [13]. Among the targeted amino acid residues, lysine 992 appears to be covalently attached to UMP [15]. When serine 948 and threonine 977 are replaced by phenylalanine and alanine respectively, CPSase become insensitive to UMP, but is still activated by ornithine [16, 17]. Because the tertiary structures and catalytic mechanisms of CPSase from *B*. *subtilis* are similar to those from other species, mutations of P1016L and E949*, which are located within the allosteric regions of *E*.*coli* CPSase, may have positive effects on the enzyme regulation. To test this hypothesis, the fragment including the mutant bases was amplified from mutant A219 and A260, respectively, and introduced into the *pyrAB* gene of the parent strain TD 12np by the method of homologous recombination [38]. High concentration of 6-azauracil was used for transformants selection. The resultant strain TD 12np-*pyrAB* ^P1016L^ and TD 12np-*pyrAB* ^E949*^ showed significantly enhanced tolerance to UMP analogues (Table 1). This suggested that mutations of P1016L and E949* on the large subunit of *B*. *subtilis* CPSase was of importance for the resistance to UMP inhibition.

In addition, it is worth noting that despite the gene sequence of *pyr* operon in mutant A260, A566 and F126 was the same, the uridine titer and yield of these mutants was different. This indicated that genomic variation happened in the pyrimidine biosynthesis- related pathway, which may also influence the uridine production. Further work may focus on the global analysis of genetic and metabolic mechanisms resulting in uridine over-production.

## Conclusions

ARTP mutagenesis coupled with high throughput screening method was performed in this study to improve uridine production. Four mutants were selected from numerous mutants and exhibited good uridine-production and genetic stability. Mutant *B*. *subtilis* F126 accumulated 30.3 g/L uridine in 48 h fed-batch fermentation, 8.7-fold higher than the parent strain. Sequence analysis of the four mutants showed that proline 1016 and glutamate 949 in the large subunit of *B*. *subtilis* CPSase may be associated with the binding and allosteric regulation by UMP and other effectors.

## Supporting information

S1 Fig Uridine standard curves established by microplate reader at a detection wavelength of 270nm. (TIF)

S2 Fig Lethality rate of B. subtilis under different irradiation time. (TIF)

S3 Fig Uridine titer of mutants isolated from 2-thiouracil-resistance plate. (a) 200 mg/L 2-thiouracil; (b) 300 mg/L 2-thiouracil; (c) 400 mg/L 2-thiouracil.
(TIF)

S4 Fig Uridine titer of mutants isolated from 6-azauracil-resistance plate. (a) 100 mg/L 6-azauracil; (b) 3 g/L 6-azauracil.
(TIF)

S5 Fig Uridine titer of mutants isolated from 200 mg/L 5-fluorouracil-resistance plate. (TIF)

S1 Table Primers for sequence analysis and site mutagenesis. (PDF)
