# Supplementary figures and images for: Improvement of uridine production of Bacillus subtilis by atmospheric and room temperature plasma mutagenesis and high-throughput screening
Source: PLoS One. 2017 May 4;12(5):e0176545. doi: 10.1371/journal.pone.0176545 (PMC5417507; doi:10.1371/journal.pone.0176545)

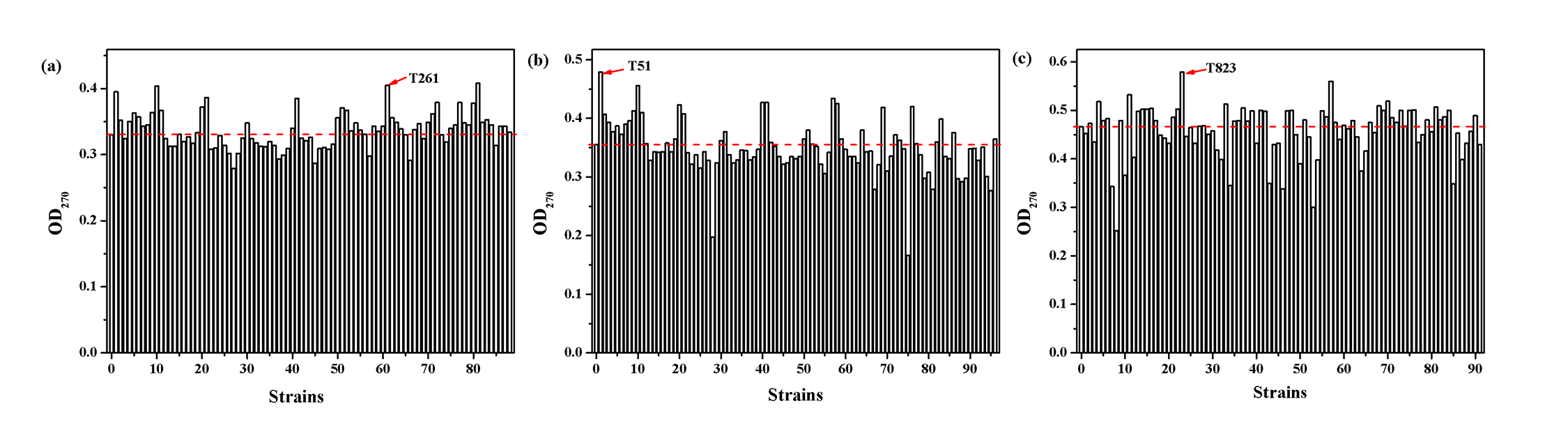

Supplement: S3 Fig — (a) 200 mg/L 2-thiouracil; (b) 300 mg/L 2-thiouracil; (c) 400 mg/L 2-thiouracil. (TIF) [file pone.0176545.s003.tif]

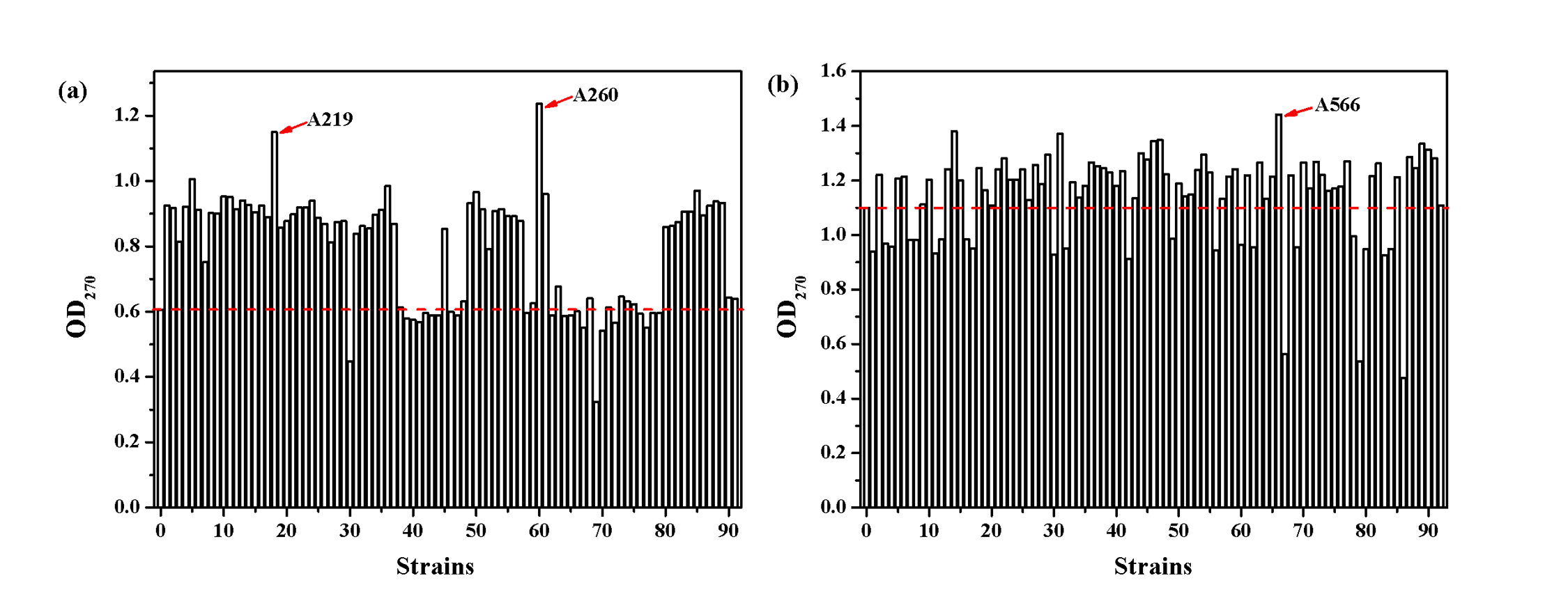

Supplement: S4 Fig — (a) 100 mg/L 6-azauracil; (b) 3 g/L 6-azauracil. (TIF) [file pone.0176545.s004.tif]
