# Supplementary material for: Improvement of uridine production of Bacillus subtilis by atmospheric and room temperature plasma mutagenesis and high-throughput screening
Source: PLoS One. 2017 May 4;12(5):e0176545. doi: 10.1371/journal.pone.0176545 (PMC5417507; doi:10.1371/journal.pone.0176545)
Supplement: S1 Table — (PDF) [file pone.0176545.s006.pdf]

**S1 Table. Primers for sequence analysis and site mutagenesis.**

| Primers 5’ → 3’ | Characteristics |
|---|---|
| ***pyrAA-s*** | AACAGCATTCCCGCTTCTTTAC |
| ***pyrAA-a*** | CTCTTTCAGAAAGCTCAACCGC |
| ***pyrAB1-s*** | TGAAAAAATGAAATTCGGCCAC |
| ***pyrAB1-a*** | CAGTGATCGCACTGCCTTCAG |
| ***pyrAB2-s*** | AAAACATATGCAGCATTTGAACCT |
| ***pyrAB2-a*** | GCACGTTCAATGTGCTCCATAA |
| ***pyrAB3-s*** | TACCATGAAGAGGAACTGCTTCATT |
| ***pyrAB3-a*** | TAACAGGAAAGCCATTTCCCAA |
| ***pyrC-s*** | CGTAGAACGGGCTGAGCGTA |
| ***pyrC-a*** | AAAATCATCACGGTTAATGCCG |
| ***pyrD-s*** | ACACCGATGCTCAAGGCGT |
| ***pyrD-a*** | CAAGGCGCTTCATCGCTTT |
| ***pyrE-s*** | AAAGCCATTTACCAAGCGGTG |
| ***pyrE-a*** | TATGCAGGTTGCCGGGCT |
| ***pyrF-s*** | CCCGATTATCGGAATGGGAG |
| ***pyrF-a*** | ATCATTTCAGCTTCAGGAAAATGC |
| ***pyrK-s*** | TTTGTCATCAATACGCTGACAAAAG |
| ***pyrK-a*** | ATTGAGCATTCCAGCACCAGTC |
| ***pyrAB*** $^{\mathbf{P1016L}}$***-s*** | TACCATGAAGAGGAACTGCTTCATT |
| ***pyrAB*** $^{\mathbf{P1016L}}$***-a*** | TAACAGGAAAGCCATTTCCCAA |
| ***pyrAB*** $^{\mathbf{E949*}}$***-s*** | TACCATGAAGAGGAACTGCTTCATT |
| ***pyrAB*** $^{\mathbf{E949*}}$***-a*** | TAACAGGAAAGCCATTTCCCAA |
